# An Internet-Based Intervention to Increase the Ability of Lesbian, Gay, and Bisexual People to Cope With Adverse Events: Single-Group Feasibility Study

**DOI:** 10.2196/56198

**Published:** 2024-05-15

**Authors:** Andreea Bogdana Isbășoiu, Florin Alin Sava, Torill M B Larsen, Norman Anderssen, Tudor-Stefan Rotaru, Andrei Rusu, Nastasia Sălăgean, Bogdan Tudor Tulbure

**Affiliations:** 1 Department of Psychology West University of Timisoara Timisoara Romania; 2 Department of Psychology and Educational Sciences Transilvania University of Brasov Brasov Romania; 3 Department of Health Promotion and Development University of Bergen Bergen Norway; 4 Department of Psychosocial Science University of Bergen Bergen Norway; 5 Department of Bioethics University of Medicine and Pharmacy “Gr. T. Popa” Iași Iasi Romania

**Keywords:** acceptance and commitment therapy, anxiety, depression, PTSD, LGBTQ+, online interventions, transdiagnostic, prevention

## Abstract

**Background:**

Lesbian, gay, bisexual, transgender, and queer (LGBTQ+) people are at higher risk of mental health problems due to widespread hetero- and cisnormativity, including negative public attitudes toward the LGBTQ+ community. In addition to combating social exclusion at the societal level, strengthening the coping abilities of young LGBTQ+ people is an important goal.

**Objective:**

In this transdiagnostic feasibility study, we tested a 6-week internet intervention program designed to increase the ability of nonclinical LGBTQ+ participants to cope with adverse events in their daily lives. The program was based on acceptance and commitment therapy principles.

**Methods:**

The program consists of 6 web-based modules and low-intensity assistance for homework provided by a single care provider asynchronously. The design was a single-group assignment of 15 self-identified LGB community members who agreed to participate in an open trial with a single group (pre- and postintervention design).

**Results:**

Before starting the program, participants found the intervention credible and expressed high satisfaction at the end of the intervention. Treatment adherence, operationalized by the percentage of completed homework assignments (32/36, 88%) was also high. When we compared participants’ pre- and postintervention scores, we found a significant decrease in clinical symptoms of depression (Cohen *d*=0.44, 90% CI 0.09-0.80), social phobia (*d*=0.39, 90% CI 0.07-0.72), and posttraumatic stress disorder (*d*=0.30, 90% CI 0.04-0.55). There was also a significant improvement in the level of self-acceptance and behavioral effectiveness (*d*=0.64, 90% CI 0.28-0.99) and a significant decrease in the tendency to avoid negative internal experiences (*d*=0.38, 90% CI 0.09-0.66). The level of general anxiety disorder (*P*=.11; *d*=0.29, 90% CI –0.10 to 0.68) and alcohol consumption (*P*=.35; *d*=–0.06, 90% CI –0.31 to 0.19) were the only 2 outcomes for which the results were not statistically significant.

**Conclusions:**

The proposed web-based acceptance and commitment therapy program, designed to help LGBTQ+ participants better manage emotional difficulties and become more resilient, represents a promising therapeutic tool. The program could be further tested with more participants to ensure its efficacy and effectiveness.

**Trial Registration:**

ClinicalTrials.gov NCT05514964; https://clinicaltrials.gov/study/NCT05514964

## Introduction

### Overview

Social inclusion of lesbian, gay, bisexual, and transgender (LGBT) individuals varies within and between countries (eg, measures of public acceptance of homosexuality and same-sex family rights) [1]. However, the LGBT groups are, on average, at a higher risk for mental health problems [2,3] as a result of various types of marginalization and discrimination working through processes of minority stress [4]. This includes repeated experiences of adverse social events that can lead to internalized homo-, bi-, and transphobia. For example, the vulnerability of gay men to developing symptoms of depression and anxiety is explained by the stress of being part of the lesbian, gay, bisexual, transgender, and queer (LGBTQ+) community. This type of stress includes experiences of discrimination and bullying, as well as the internalization of negative social attitudes. However, some research suggests that promoting self-acceptance may be beneficial in reducing self-stigma related to sexual orientation. This study examined how a specially tailored internet intervention program might help improve self-acceptance among LGB individuals.

### Scientific Background

The literature in this area includes studies of various types of interventions conducted to support the LGBTQ+ community, such as a camp intervention for LGBTQ+ youth to reduce depressive symptoms [5], a cognitive behavioral therapy (CBT) intervention for body image, and self-care for gay men living with HIV [6], an affirmative CBT intervention for depression caused by sexual orientation discrimination [7], an identity-affirming web app to cope with minority stress [8], an intensive outpatient group program tailored form LGBTQIA+ to reduce depression and anxiety [9], a rejection sensitivity model used to extend minority stress theory to improve mental health [10], and even a socially assistive robot to be used by young LGBTQ+ at risk for self-harm [11]. All of these and others, including this study, have been designed and implemented by psychologists who have shown an interest in improving the quality of life for the LGBTQ+ community.

Digital interventions such as those delivered through web platforms or smartphones are highly relevant for the LGBTQ+ population, as many people prefer to conceal their identity, particularly in countries with high levels of homophobia and transphobia. A recent systematic review [12] on digital health interventions for LGBTQ+ participants concluded that there were more interventions aiming at reducing sexually transmitted diseases than for other health concerns and that more targeted interventions are needed to cover mental health difficulties.

This study aims to design and preliminary test a stand-alone digital intervention tailored for LGBTQ+ participants that originates from the acceptance and commitment theory [13-15]. Several studies have emphasized the protective role of psychological processes related to acceptance. Acceptance implies psychological flexibility, from which 2 essential resources are derived [13-15]. One resource is the ability to accept the experience of the present moment as it is rather than avoid unpleasant events. A crucial rule is to accept the diversity of emotional experiences, not just the positive ones. One strategy for achieving this goal is to view unpleasant events as external events rather than overidentifying with these difficulties and blaming oneself. While avoidance is a coping strategy often used by LGBTQ+ people to deal with adverse events [16], there is also evidence to suggest that this emotional regulation strategy is associated with poor mental health [17]. Experiential avoidance, viewed by Hayes et al [18] as the opposite of acceptance, is a stronger predictor of depression than internalized homophobia. It also mediates the relationship between internalized homophobia and the severity of depressive symptoms [19]. In a recent systematic review, there was a call for action for researchers to provide evidence for the effectiveness of acceptance and commitment therapy (ACT) in treating mental health issues expressed by the LGBT community [20] in the absence of methodologically robust studies on this topic. An exception outside the ACT area [21] provided evidence for the effectiveness of an online single session in reducing internalized stigma and slightly increasing identity pride. Increasing the number of sessions from 1 to 6 and focusing on ACT principles to increase self-acceptance may increase the effectiveness of an online intervention tailored to the mental health needs of LGBTQ+ adults.

### Goals of This Study

The main objective was to design and implement an internet-delivered (digital) psychological program based on ACT principles to help LGBTQ+ individuals become more resilient.

This study (NCT05514964) had three aims: (1) to tailor a prevention program based on ACT principles to the specific needs of the LGBTQ+ community; (2) to assess the feasibility of the program operationalized as treatment acceptability and treatment satisfaction among participants; and (3) to preliminary test the impact of the intervention program on participants’ levels of psychological flexibility, anxiety, and depression. Together with an increased sense of personal agency—that is, the ability to make changes in one’s life and control one’s destiny—we expect that LGBTQ+ individuals will be better equipped to cope with potential adverse events, including discrimination. The premises for an online intervention for LGBTQ+ participants are favorable because such interventions are more appropriate for LGBTQ+ people who are closeted and can cover more geographical locations, including areas outside of major cities where face-to-face psychological services are less available.

We hypothesized that the ACT-based mental health prevention program tailored for the specific needs of the LGBTQ+ community would be perceived as being (1) credible (logical) in comparison with a neutral point on a not logical–very logical continuum and (2) will lead to beneficiaries’ satisfaction in comparison with a neutral point on a totally unsatisfied–very satisfied continuum. Likewise, we hypothesized significant improvements in the (3) level of anxiety, (4) social anxiety, (5) depression, and (6) alcohol consumption between the pretest and posttest as evidence in favor of treatment feasibility for these primary outcomes.

## Methods

### Recruitment and Procedure

Participants were recruited using a variety of advertising methods targeting the LGBTQ+ community. We used online postings supported by several local NGOs (Accept, MozaiQ, Identity Education, and Campus Pride). The official description of the program was posted on the project website and social media (Facebook [Meta] and Grindr [Joel Simkhai]), and posters were printed for LGBTQ+ nightclubs. A brief description of the program was included in a newsletter, and one of the authors presented the program to potentially interested participants during a Campus Pride event.

Participants were invited to log in to a psychotherapy platform using a personal email account to participate in the study. Before registering, participants were encouraged to create a new email account to maintain anonymity. Registered participants were asked to complete a series of screening questionnaires to assess their eligibility. Based on these descriptions, computer and internet literacy was expected from participants in the study.

Treatment credibility was also assessed before the program. Inclusion criteria were being older than 18 years; fluent in Romanian; and being gay, lesbian, bisexual, or transgender. All eligible participants in the study should also have low or moderate symptoms on at least one of the following self-report scales: General Anxiety Disorder-7 (GAD-7; a score between 5 and 14) for generalized anxiety, Social Phobia Inventory (SPIN; a score between 21 and 40) for social anxiety, Patient Health Questionnaire (PHQ-9; a score between 5 and 14) for depression, and Alcohol Use Disorders Identification Test (AUDIT; a score between 8 and 14) for alcohol use. The exclusion criteria were suicidal ideation (ie, exceeding a score of 1 on suicide item 9 from PHQ-9), changes in psychotropic medication dosage in the past month (if present), bipolar disorder or psychosis (according to medication status), severe alcohol abuse or dependence (AUDIT score ≥15), clinical levels of anxiety or depression (exceeding the previously mentioned thresholds), current participation in other psychological treatment, and an obvious barrier to participation (eg, no current internet access, extended travel plans during the treatment period).

### Procedure

Enrolled participants were asked to read the first intervention module and complete homework assignments. No bug fixes or unexpected events occurred in the functionality of the platform. Finally, after the 6-week intervention, participants were invited to complete the postintervention assessment measures, which self-assessed their levels of anxiety, depression, and alcohol use through online questionnaires. Treatment satisfaction data were collected after the intervention to measure whether participants were satisfied with the intervention through online self-assessed questionnaires.

### Participants

While the intervention was active online, 169 individuals expressed interest in the study by accessing the web platform. Of these, 65 completed all or most of the screening questionnaires, and 15 were eligible for inclusion in the study (Table 1 shows the demographics). All these 15 participants successfully completed the program.

**Table 1:** The demographic characteristics of included participants.

Demographic variables		Value
**Age (years)**		
	Mean (SD)	29.86 (9.53)
	Range	21-50
**Biological sex, n (%)**		
	Female	10 (66.7)
	Male	5 (33.3)
**Gender identity, n (%)**		
	Woman	10 (66.7)
	Man	5 (33.3)
	Nonbinary	0 (0)
**Sexual orientation, n (%)**		
	Homosexual	6 (40)
	Bisexual	9 (60)
**Coming out, n (%)**		
	Undisclosed identity	3 (20)
	Revealed to some	7 (46.7)
	Revealed to all	5 (33.3)
**Marital status, n (%)**		
	Never married	10 (66.6)
	Married	1 (6.7)
	In a relationship	4 (26.7)
**Professional status, n (%)**		
	Student	5 (33.3)
	Part-time	1 (6.7)
	Full-time	8 (53.3)
	Unemployed	1 (6.7)

### The Program

The current prevention program is based on a previously tested intervention based on ACT principles [22] and reported according to the CONSORT-EHEALTH (Consolidated Standards of Reporting Trials of Electronic and Mobile Health Applications and Online Telehealth) checklist [23]. However, this intervention was initially designed for the general population, and the original version of the program did not include specific references to sexual orientation. Therefore, our team decided to make the program LGBTQ+ friendly and tailor the content to the particular needs of this community. The tailoring process was theoretically informed by the American Psychological Association’s general recommendations for psychological practice with lesbian, gay, and bisexual clients [24]. These guidelines provide a general framework for psychological services with the LGBTQ+ community, systematically addressing the important issues and pertinent features that may arise in this context. We have also incorporated other suggestions from the literature [25,26], where comparable interventions have been successfully tested with the LGBTQ+ community. Finally, we thoroughly discussed the tailoring process and the new content to be added to the program with several members of the local LGBTQ+ communities. Some of these members were actively involved in various local LGBTQ+ organizations, some had professional training in psychology, and some were community members. The tailoring process took about 6 months, during which feedback was collected, discussed, and ultimately incorporated into the final version of the program. As a result, 6 customized modules adapted for the online environment were finally uploaded to a psychotherapy web platform.

The 6 modules covered six different topics: (1) introduction to minority stress and the current program, (2) defusion, (3) coming out and the acceptance process, (4) personal values, (5) committed action, and (6) compassion and self in context. The recommended time for each module was 1 week, so the total length of the program was 6 weeks. Participants were asked to complete approximately 6 homework assignments per module (36 for the entire program). At the end of each week, an online therapist provided written feedback to each participant on the platform. To ensure confidentiality, the content of the messages was securely stored on encrypted software. Participants received only a notification when a new written message was delivered to them within the psychotherapy platform, but their registered email was not used to provide any sensitive data. The content of the 6 modules is available for replicability or upscaling studies at the LGBT Inclusion website [27]. Human involvement was limited to providing asynchronous written feedback and reminders for homework. The assistance was provided by a single care provider (research assistant).

### Treatment Credibility, Satisfaction, and Adherence Measures

Participants were asked to complete a measure of treatment credibility before the program began and a measure of treatment satisfaction immediately after the intervention to assess their overall perceptions of the program. A total of 5 standard questions on operationalized treatment credibility were scored on a 10-point Likert scale (ie, the program: 0=does not seem logical to 10=seems very logical). After the intervention, we assessed participants’ satisfaction with the program by asking them to complete a series of quantitative and qualitative items, that is, overall satisfaction with treatment (1=totally unsatisfied vs 5=very satisfied). More details on treatment credibility and satisfaction questions are presented in the Results section.

Treatment adherence (the intensity or dose of the intervention) was estimated by the number of homework assignments completed for each participant.

### Outcome Measures

#### Primary Outcome Measures

The SPIN [28] was designed to measure the participant’s level of social phobia. The scale is unidimensional, and the total score ranges from 0 to 68, with high scores associated with high levels of social phobia. In this study, the internal consistency was adequate α=.87.

The PHQ-9 [29] was designed to measure the participant’s level of depression, with high scores associated with high levels of depression. In this study, the internal consistency was α=.84.

The GAD-7 [30] was designed to measure the participants’ level of anxiety or worry. The total score ranges from 0 to 21, with high scores associated with high levels of worry. The instrument has demonstrated adequate psychometric properties [31,32], and in our sample, the internal consistency was α=.87.

The AUDIT [33] consists of 10 items measuring alcohol use. In this study, internal consistency for this scale was α=.73.

#### Secondary Outcome Measures

The Acceptance and Action Questionnaire 2 [34] assessed psychological flexibility. In this study, the internal consistency was α=.81.

The Brief Multidimensional Experiential Avoidance Questionnaire [35] was designed to measure the participants’ avoidance tendencies. The internal consistency in this study was α=.84.

The Diagnostic and Statistical Manual-5 Post-Traumatic Stress Disorder Checklist (PCL-5) [36] was used to measure symptoms of posttraumatic stress disorder (PTSD). For this study, the internal consistency of the PCL-5 was (α=.91).

Additional measures were used during the screening process related to how participants experience their lives as members of the LGB community. These three additional measures were (1) the Short Internalized Homonegativity Scale [37], (2) the Sexual Orientation Concealment Scale [38], and (3) the Daily Heterosexist Experiences Questionnaire [39].

### Ethical Considerations

The study was approved by the Ethics Committee of the West University of Timisoara, Romania (4137/27.01.2021) and was registered on ClinicalTrials.gov as NCT05514964. Written informed consent was obtained from all participants by surface email.

## Results

### Treatment Credibility, Satisfaction, and Adherence

Table 2 shows the mean scores for the included participants. The scores for all items were significantly above chance (set at 5 on the 10-point scale, all *P*<.001), as participants reported that the program seemed trustworthy.

As shown in Table 3, participants completed most of the modules and seemed satisfied with the intervention. The fact that most participants remained active throughout the program and completed the postintervention assessment proves that the program was perceived as useful despite the time (3.6 hours per week) and effort required to complete it.

Treatment adherence (the intensity or dose of the intervention) was estimated by the number of homework assignments completed for each participant. On average, participants completed 5.3 weekly assignments (out of a maximum of 6). More specifically, the 15 participants completed, on average, 32 out of a maximum of 36 assignments (88%), representing high adherence.

**Table 2:** Descriptive statistics for the treatment credibility items.

Assessed dimension	Mean (SD)
Intervention program seems logical	9.06 (1.03)
Confidence that the program will be helpful	7.93 (2.01)
Confidence in recommending the program to a friend	7.93 (2.6)
Program seems effective in helping me manage my emotions	8.20 (1.85)
I expect to be able to manage my emotions by the end of the program	7.93 (1.38)

**Table 3:** Descriptive statistics for the quantitative items included in the treatment satisfaction assessment.

The overall satisfaction with the treatment	Mean (SD)
Overall satisfaction with treatment (1=totally unsatisfied vs 5=very satisfied)	4.46 (0.51)
Quality of information provided (1=very poor vs 5=very good)	4.53 (0.74)
Satisfied with intervention timing (1=too short, 3=appropriate, and 5=too long)	2.53 (0.64)
Number of completed modules (out of 6)	5.80 (1.08)
Number of fully grasped modules (out of 6)	5.46 (1.24)
The average time spent with the program (hours per week)	3.60 (1.99)
Program demandingness (1=not demanding vs 4=very demanding)	2.77 (0.7)
The program helped me approach problems more effectively (1=not at all vs 4=to a great extent)	3.4 (0.5)

### Treatment Outcomes

Subsequently, we assessed the impact of the program by comparing participants’ levels of anxiety, depression, and alcohol use before and after completing the program. Table 4 shows us that participants improved on most outcome measures, except the AUDIT and GAD-7. Participants’ alcohol consumption seems to remain unaffected by the program while their anxiety and depression levels decrease. We also found that participants’ psychological flexibility increased significantly (Cohen *d*=0.83).

We found a significant improvement in participants’ psychological flexibility (Acceptance and Action Questionnaire 2, Cohen *d*=0.64), the primary goal of any ACT-based intervention. We also found significant reductions in clinical symptoms of depression (*d*=0.44), social phobia (*d*=0.39), and PTSD (*d*=0.30). However, participants’ levels of general anxiety disorder and alcohol use were the only 2 outcomes for which the results were not statistically significant.

**Table 4:** Means and SDs before and after the psychological intervention.

Measures	Pretreatment, mean (SD)	Posttreatment, mean (SD)	Student’s *t* test (*df*)	*P* value (1-tailed)	Cohen *d* (90% CIs)
AUDIT^a^	3.66 (3.35)	3.86 (3.37)	–0.40 (14)	.35	–0.06 (–0.31 to 0.19)
PHQ-9^b^	10.86 (6.04)	8.26 (5.67)	2.17 (14)	.02	0.44 (0.09 to 0.80)
GAD-7^c^	9.26 (5.25)	7.60 (6.03)	1.25 (14)	.11	0.29 (–0.10 to 0.68)
SPIN^d^	25.00 (11.17)	20.26 (12.61)	2.13 (14)	.02	0.39 (0.07 to 0.72)
AAQ-2^e^	40.80 (7.81)	46.20 (8.93)	–3.23 (14)	.003	0.64 (0.28 to 0.99)
B-MEAQ^f^	48.80 (9.70)	44.53 (12.03)	2.24 (14)	.02	0.38 (0.09 to 0.66)
PCL5^g^	34.13 (14.17)	27.40 (22.34)	1.99 (14)	.03	0.30 (0.04 to 0.55)

^a^AUDIT: The Alcohol Use Disorders Identification Test.

^b^PHQ-9: Patient Health Questionnaire-9.

^c^GAD-7: Generalized Anxiety Disorder-7.

^d^SPIN: Social Phobia Inventory.

^e^AAQ-2: Acceptance and Action Questionnaire 2.

^f^B-MEAQ: Brief-Multidimensional Experiential Avoidance Questionnaire.

^g^PCL5: Posttraumatic Stress Disorder Checklist for Diagnostic and Statistical Manual-5.

## Discussion

### Principal Results

In this study, we examined the feasibility of an internet-delivered prevention program tailored for LGBTQ+ individuals at risk for developing emotional disorders due to minority stress (eg, being discriminated against, not being accepted by their families, etc). The intervention was designed to increase personal agency and foster acceptance among LGB individuals. Overall, the intervention was perceived as credible based on participants’ involvement in the program and generated high levels of satisfaction at the end of the program. Participants also demonstrated a high level of treatment adherence, completing 88% (32/36) of the homework assignments. In terms of impact, we found significant reductions in clinical symptoms of depression, social phobia, and PTSD following the intervention. There was also a substantial improvement in self-acceptance and a considerable decrease in the avoidance of negative experiences.

This was the first online transdiagnostic prevention program targeting the LGB group based on the ACT framework. Despite previous calls to use standard ACT [40] or affirmative ACT [41] for LGBTQ+ communities, this is the first ACT-inspired open trial tailored for LGBTQ+ people. It is not, however, the first mental health program tailored to the LGBTQ+ community. For example, Pachankis et al [26] used a 10-session LGBTQ+ tailored CBT. They found reductions in a wide range of symptoms such as depression, anxiety, and co-occurring health risks (ie, alcohol use and condomless sex) among young adult gay and bisexual men. It should be noted, however, that this intervention was based on face-to-face sessions with experienced CBT therapists.

Considering the privacy issues, the shortage of trained mental health professionals in Romania and internationally, and the fact that even those who exist may not have sufficient knowledge or experience working with sexual minorities, an internet-delivered program looks pretty suitable for LGBTQ+ participants. They are more reluctant to participate in such programs, preferring to hide their identity. The challenge is to make evidence-based interventions available to underserved populations, such as LGBTQ+ people with emotional difficulties.

Internet-delivered or computerized interventions may address the above limitations by becoming an attractive alternative in a stepped-care approach for those seeking treatment for mild to moderate symptoms [42]. The internet-based treatment format has the potential to reduce many of the barriers that currently impede access to mental health care: the small number of competent psychotherapists, geographic distance, and the high cost of face-to-face programs. However, many internet-delivered studies have not been implemented for the LGBTQ+ community, with one exception: the computerized CBT-Rainbow SPARX (Smart, Positive, Active, Realistic, X-Factor Thoughts), aimed at reducing depression symptoms in adolescents who manifested same-sex attraction [43]. However, the program was designed primarily for adolescents in a gamified form. Participants had to use avatars to collect all 6 gems for a shield. This strategy limits its applicability to adults, who are less likely to participate in a gamified psychosocial intervention.

Mental health issues in the LGBTQ+ community are multifaceted and have a significant impact on the psychological and physical well-being of those affected. Several studies have documented that people who are part of the LGBTQ+ community are vulnerable to various status inequalities related to income disparities, treatment in the workplace, and social and legal discrimination [44,45,46], as same-sex unions are not recognized. Targeting and changing such societal processes requires several long-term structural changes. An online intervention program targeting personal acceptance, identity, engagement, and coping strategies is only one of several strategies to reach the LGB and LGBTQ+ communities.

### Limitations

This feasibility study provided us with experience in making relevant changes in different parts of the procedures and the program. First, we observed that several potential participants did not join the program because of too many measurements, including too many instruments that asked in great detail about the participants’ lives and intruded too much into their privacy, which scared them, considering that we are in a country where this category of the population is very discriminated against. To diminish the risk that the research process itself could be seen as tiring and intrusive, as it involves revealing aspects of their lives that are stigmatized, we suggest dropping out some measures to reduce the burden of participants and advertising the call to potential beneficiaries only after establishing a direct contact with them to decrease their mistrust level. Second, the study did not include follow-up measurements. Future studies should include follow-up measurements to decide whether the intervention has significant long-term effects. This would involve providing incentives for their long-term participation, including sharing the progress or preliminary results, to help participants notice their contribution is meaningful and valuable. Third, as with any feasibility study, an underpowered study undermines some potential conclusions. It may be that relying solely on increasing the sample size would be sufficient to decrease the generalized anxiety symptoms significantly. Or perhaps an extension from a 6-week to a 9-week program would be required to reduce the anxiety symptoms significantly. Similarly, the current program focuses extensively on internalizing problems, so a program redesign is needed when targeting externalizing problems such as alcohol use.

### Conclusions

The 6-week online transdiagnostic program based on ACT, specifically designed for the LGBT community, is a promising intervention because the treatment is credible and results in beneficiaries’ satisfaction. In addition, the dynamics of the pre- and posttest data suggest clinical improvement in most of the measured outcomes, including depression, PTSD, and social phobia, as well as in the potential related mechanisms of change from an ACT perspective. The online program is particularly appropriate for use in countries where beneficiaries are less likely to disclose themselves and where there is a shortage of therapists due to geographic or other barriers.

## Abbreviations

ACT: acceptance and commitment therapy
AUDIT: Alcohol Use Disorders Identification Test
CBT: cognitive behavioral therapy
CONSORT-EHEALTH: Consolidated Standards of Reporting Trials of Electronic and Mobile Health Applications and Online Telehealth
GAD-7: Generalized Anxiety Disorder
LGBT: lesbian, gay, bisexual, and transgender
LGBTQ+: lesbian, gay, bisexual, transgender, and queer
PCL-5: Diagnostic and Statistical Manual-5 Posttraumatic Stress Disorder Checklist
PHQ-9: Patient Health Questionnaire
PTSD: posttraumatic stress disorder
SPARX: Smart, Positive, Active, Realistic, X-Factor Thoughts eCBT
SPIN: Social Phobia Inventory
